# Editorial: The Use of Post-exercise Cooling as a Recovery Strategy: Unraveling the Controversies

**DOI:** 10.3389/fspor.2022.825016

**Published:** 2022-02-24

**Authors:** Mohammed Ihsan, Chris R. Abbiss, Robert Allan

**Affiliations:** ^1^Human Potential Translational Research Program, Yong Loo Lin School of Medicine, National University of Singapore, Singapore, Singapore; ^2^Centre for Exercise and Sports Science Research, School of Medical and Health Sciences, Edith Cowan University, Perth, WA, Australia; ^3^School of Sport and Health Sciences, University of Central Lancashire, Preston, United Kingdom

**Keywords:** cold water immersion, cryotherapy, phase change material, physiological recovery, muscle strength

## Introduction

Post-exercise cooling is a popular recovery strategy utilized by athletes, and of interest to many research groups. Significant body of research have examined the effects of post-exercise cooling on outcomes such as physical performance, regulation of inflammatory biomarkers, and psychophysical indices related to perceived fatigue, recovery and wellbeing. Despite the research and widespread use by athletes, there is considerable skepticism regarding its efficacy, in-part stemming from mixed findings reported within the literature. Moreover, emerging work demonstrating dampened hypertrophy gains following the regular use of cold-water immersion (CWI) has further questioned the appropriateness of cooling modalities as a recovery technique. Many of these diverse findings may be reconciled through considering factors such as the cooling modality and protocol, nature of exercise stressor, and type and timing of recovery assessment. Regardless, controversy remains with regards to the use of post-exercise cooling, often resulting in mixed and misinformed messages for practitioners in applied settings.

Accounting for and understanding the differences within experimental context is critical for the appropriate use of post-exercise cooling in applied practice. In this Research Topic, we invited scientists to offer commentary and critically examine key areas surrounding the use of recovery cooling strategies. This will aid in developing the available body of literature into appropriate context for practitioners and provide directions for future research. Thirteen articles were accepted for publication (five original research, three reviews, two mini-reviews, two opinion pieces, and one systematic review), written by a total of 53 contributing authors. We have summarized and discussed this collection with regards to improving applied practice, as well as addressing controversies surrounding the use of post-exercise cooling.

## Cold Water Immersion and Strength Adaptation

Petersen and Fyfe reviewed the influence of CWI on strength adaptation. The authors concluded that whilst CWI may enhance short-term recovery following resistance exercise, the majority of the evidence aligned toward dampened molecular signatures and physiological adaptations to resistance training.

Conversely, Tavares et al. showed no impairments, in fact demonstrated a trend toward improved vertical jump performance following regular use of CWI (i.e., 12 sessions of CWI over 3 weeks) during intensified training. In support, review by Ihsan et al. highlighted other works showing no impairments, instead a trend toward improved strength amongst athletes undertaking post-exercise CWI over 2.5 weeks−8 months. This disconnect between Petersen and Fyfe and others (Tavares et al.; Ihsan et al.) may be reasoned by differences in experimental approach, where laboratory-based studies have typically employed 2–3 training sessions per week, potentially allowing for full recovery between sessions. Conversely, in applied scenarios where recovery between training sessions is limited (i.e., >10 sessions/week), CWI appears to improve subsequent training performances and consequently allow maintenance of a sufficient training stimulus for adaptation. Moreover, beneficial recovery outcomes of CWI may be harnessed by programming this modality following technical or aerobic conditioning, and avoiding it following resistance training sessions (Ihsan et al.).

Recovery periodization thus has been proposed within this Research Topic, where post-exercise cooling is strategically programmed to align with the suitability of the preceding exercise task, the physiological system(s) that requires restoration, and the need for recovery dictated by changes in training demands and athlete wellness (Tavares et al.; Ihsan et al.; Thorpe).

## Cooling Between High-Intensity Efforts

Egaña et al. investigated the effects of 5–10 min CWI administered between 2 bouts of 4 km cycling time-trials performed in normothermia. The authors reported similar performances compared to passive or active recovery, despite alleviated thermal and perceptual strain following CWI. The mechanisms by which CWI may benefit such performance tasks are not clear, but plausible avenues include haemodynamic changes (influencing venous return), cardiac parasympathetic reactivation and/or reduced perception of effort. Regardless, the use of cooling amidst closely scheduled high-intensity and short duration efforts is at best contentious, especially in the absence of environmental heat stress. This is gathered from long-standing evidence supporting warmer temperatures for improved muscle function, and hyperthermia not being a primary limiting factor during such short task. This perhaps reiterates to first identify the origins of fatigue and the physiological system(s) that need recovery, which then will serve as the driving factor to select appropriate recovery intervention(s) (Thorpe).

## Post-Exercise Cooling and Perceptual Recovery

L'Hermette et al. explored how CWI can influence sensorial experiences and accompanying physiological recovery. CWI alleviated muscle soreness, facilitated sympathetic withdrawal and increased parasympathetic activity. Although athletes reported of pain and discomfort upon immersion, these sensations progressively declined over the initial 3 min, and was superseded by improved sense of relaxation, wellbeing, vigor, and vitality. Future work should investigate how such sensorial experiences evolve over intensified training periods. Importantly, it is critical to understand how such changes translate to improved physical function, given that Lindsay and Peake have identified muscle strength and/or power as a direct, training-specific variable encompassing recovery.

## Individualizing and Optimizing Cooling Protocols

Work within this Research Topic (Freitag et al.) and elsewhere (Stephens et al., 2018) have provided typical muscle and core temperature changes following common CWI protocols. Practitioners can utilize these resources in gathering estimates of body temperature changes, given that regular measurements are impractical in applied settings involving athletes. While such resources are useful, individualizing temperature thresholds associated with specific recovery objectives are difficult to establish, and would require occasional direct measurement of body temperatures to complement/verify estimates derived from such resources. In applied settings, Lindsay and Peake recommend regular assessments of muscle strength and/or power, as they provide specific, interpretable data for practitioners and coaches. Regular performance measures should accompany recovery programs to ascertain the efficacy of a particular cooling modality or protocol for a given training stimulus.

## Emerging Mechanisms

Key responses to post-exercise cooling include peripheral vasoconstriction, decrease in limb blood flow, and redistribution of blood volume from the periphery to the core. Seeley et al. highlighted that such physiological changes can help attenuate post-exercise orthostatic intolerance, amongst other associated recovery benefits. Further research is warranted to examine how cooling mitigates orthostatic intolerance triggered by exercise heat stress, and translation to physical recovery in athletes and clinical populations.

Chauvineau et al. suggested that including the head (aided by scuba kit) to CWI following evening training can improve subsequent sleep architecture. However, inclusion of head immersion decreased parasympathetic activity and hampered thermal sensation and comfort, although sleep propensity was improved. Future studies should aim to refine cooling methods/protocols to further the development of strategies to enhance sleep and recovery for athletes.

## Emerging Cooling Modalities

Other modalities featured include partial- or whole-body cryotherapy (Bouzigon et al.), battery-powered hand cooling devices (Seeley and Sherman) and specialized phase change material (PCM) (Kwiecien et al.). These modalities purportedly offer advantages over traditional methods (e.g., ice or CWI) to target various recovery objectives. For instance, prolonged (3–6 h) mild cooling stimulus conferred by PCMs are purportedly more effective to mitigate strength loss following muscle damage (Kwiecien et al.). The availability of such modalities undoubtedly allows practitioners to select the most appropriate method to elicit physiological alterations to achieve specific recovery objectives. However, possible disadvantages may include cost, energy supply and logistics when catering to multiple athletes (e.g., team sport). Further research is thus needed to solidify the evidence supporting the use of these modalities, such that associated disadvantages may be deemed secondary.

## Conclusion and Perspectives

This Research Topic has addressed controversies associated with post-exercise cooling, and have highlighted exciting avenues for further research. While this compilation is an informative resource for practitioners and scientists, formulating a recovery cooling program is an endeavor that requires purposeful trial and error, guided by experimental evidence. Among some of the pertinent questions are what physiological system(s) or performance measures are impaired? What is the natural recovery time-course of these measures? What is the nature and timing of the ensuing training activity? Is the utilized recovery assessment reliable, valid and specific? Each of these considerations will then provide the basis for integrating appropriate cooling strategies as part of the athletes' overall recovery program that is individualized and periodized.

## Author Contributions

All authors listed have made a substantial, direct, and intellectual contribution to the work and approved it for publication.

## Conflict of Interest

The authors declare that the research was conducted in the absence of any commercial or financial relationships that could be construed as a potential conflict of interest.

## Publisher's Note

All claims expressed in this article are solely those of the authors and do not necessarily represent those of their affiliated organizations, or those of the publisher, the editors and the reviewers. Any product that may be evaluated in this article, or claim that may be made by its manufacturer, is not guaranteed or endorsed by the publisher.
